# Role of Psychological Factors in Migraine

**DOI:** 10.7759/cureus.75858

**Published:** 2024-12-17

**Authors:** Xiran Yu, Ge Tan

**Affiliations:** 1 Department of Neurology, The First Affiliated Hospital of Chongqing Medical University, Chongqing, CHN

**Keywords:** anxiety sensitivity, mental disorders, migraine, pain catastrophizing, personality traits, psychological interventions

## Abstract

Migraine, marked by moderate to severe headaches, is frequently accompanied by reversible neurological symptoms. Recent studies have revealed a complex and significant relationship between psychological factors and the onset and progression of migraine. Personality traits, such as neuroticism and harm avoidance, play a crucial role in the development, progression, and treatment outcomes of migraines. Migraine patients often experience comorbid mental health conditions, such as depression and anxiety, which contribute to a diminished quality of life by exacerbating migraine-related disability and impaired occupational functioning. In particular, high levels of pain catastrophizing and anxiety sensitivity in migraine sufferers reflect their negative cognitive beliefs, which are closely linked to both their personality traits and vulnerability to mental disorders. This article explores the pathophysiological mechanisms underlying the relationship between migraine and psychological factors, including genetic influences, overlapping brain regions, 5-hydroxytryptamine (5-HT) dysfunction, and neurogenic inflammation. While traditional pharmacological treatments for migraine are often influenced by psychological factors and may have limited efficacy, psychotherapy, such as cognitive behavioral therapy and positive thinking therapy, has been increasingly recognized for its role in alleviating the psychological symptoms associated with migraine and enhancing overall therapeutic outcomes.

## Introduction and background

Migraine is a chronic neurological disorder that is characterized by episodes of moderate to severe headaches, often accompanied by nausea, vomiting, photophobia, phonophobia, and atypical cutaneous discomfort [1]. The World Health Organization (WHO) ranks migraine as the third most prevalent disease and the second most debilitating neurological disorder worldwide [2]. The annual prevalence rate of migraine is 13.5% [3]. With an estimated cost exceeding $20 billion annually, migraine represents a significant economic burden on society [4]. The etiology of migraine is complex, and the pathogenesis remains unclear. It is currently believed to be linked to altered brain function and connectivity, cortical spreading depression, a release of pro-inflammatory cytokines, and activation of the trigeminal vasculature [5]. Brain regions involved in psychological phenomena such as emotion and stress overlap partially with those involved in pain processing. These regions are also linked to imbalances in 5-hydroxytryptamine (5-HT) neurotransmission and pro-inflammatory cytokine release in the hypothalamic-pituitary-adrenal axis. These shared pathophysiological pathways suggest a close relationship between psychological factors and the onset and transmission of pain signals in the brain [6,7].

Psychological factors, primarily consisting of cognitive and emotional processes, are associated with various neurological conditions, such as epilepsy, Parkinson's disease, stroke, and Alzheimer's disease, as well as migraine [6,8]. Psychological factors are significant influences on migraine, affecting not only an individual’s emotional responses but also their perception of pain and coping strategies. For instance, anxiety, depression, stress, and negative cognitive beliefs about pain, such as pain catastrophizing, are commonly observed in migraine patients. These negative psychological factors can intensify both the frequency and severity of migraine attacks and may contribute to the chronic nature of migraines. Moreover, an individual's ability to regulate emotions and the effectiveness of cognitive interventions can play a crucial role in managing migraines [7]. Some studies have even suggested that migraine discomfort may be an "alarm" response to stress [9]. Researchers have recognized the psychological nature of migraine and the critical role of psychological factors in the pathogenesis of migraine and have classified migraine as a biopsychosocial syndrome that combines cognitive, affective, environmental, and biological factors [6,9]. The psychological nature of migraine has thus been recognized by researchers as an important factor in the development of migraine.

In recent years, research on the relationship between personality traits, mental disorders, and migraine has intensified. More notably, there has been an increased focus on the influence of cognitive beliefs, such as anxiety sensitivity and pain catastrophizing. However, the review of these studies has not been updated. This article aims to provide a concise overview of the influence of psychological factors on migraine, considering perspectives from personality traits, mental disorders, and cognitive processes.

## Review

Methods

This narrative review is based on literature retrieved from PubMed and Google Scholar databases. We conducted keyword searches using terms such as "migraine," "personality traits," "mental disorders," "emotional processing," "cognitive processes," "anxiety sensitivity," "pain catastrophizing," "pain management strategies," "psychological interventions," and "behavioral interventions." The retrieved literature covered studies related to these topics. We established clear inclusion criteria, selecting only peer-reviewed studies and excluding those with small sample sizes, unclear methodologies, or lacking data. For the studies that met the criteria, we extracted relevant information and conducted a comprehensive analysis. Finally, we integrated the main findings of the relevant studies and summarized and analyzed them based on their different results and conclusions.

Relationship between psychosocial factors and migraine

Personality Traits and Migraine

Personality traits are relatively stable patterns of thinking, feeling, and behaving across various environments and are one of the key individual differences that shape lifestyle [10].

Currently, the predominant theories of personality structure include the Psychobiological Model (PM), Eysenck's Three-Factor Model (TFM), and the Big Five Model (BFM) [11]. The PM categorizes personality into seven dimensions: four temperament dimensions (novelty-seeking, harm avoidance, reward dependence, and persistence) and three personality dimensions (self-directedness, cooperation, and self-transcendence) [11]. The TFM focuses on three primary dimensions: introversion-extraversion, neuroticism, and psychoticism [12]. The Big Five Model categorizes personality into extraversion, agreeableness, conscientiousness, neuroticism, and openness to experience [13].

Research suggests that individuals with migraines display distinct personality traits compared to the general population. Studies based on PM have found that migraine sufferers generally exhibit higher harm avoidance [14,15]. However, findings across studies are inconsistent, with some reporting no significant differences or even contradictory results [16,17]. Self-directedness also shows variable results, while other personality traits often do not significantly differ between migraine patients and non-migraine sufferers [18]. Migraine patients typically have higher scores for neuroticism than non-migraine patients [19,20] but results for extraversion and openness to experience vary [19]. Studies based on the BFM indicate that migraine patients tend to have higher levels of neuroticism, responsibility, and agreeableness, and lower levels of extraversion [21,22]. No significant differences in neuroticism or extraversion are observed in patients with or without migraine with aura [13].

Neuroticism reflects an individual's emotional stability and is associated with IL-6 levels; IL-6-induced neuroinflammation is one of the pathophysiological mechanisms linked to migraines. High harm avoidance, characterized by a pessimistic outlook and avoidant behavior, is linked to altered 5-HT function and dysfunction in transmission in the dorsal nucleus accumbens region [11,14,23]. Migraine patients have elevated levels of 5-HT in the brain during the interictal period and lower levels during migraine attacks [24]. High persistence, which manifests as determination in the face of setbacks, combined with low self-direction among migraine patients, suggests a diminished capacity to cope with stress and a heightened risk of anxiety, depression, and migraine chronicity [11]. In addition, the high neuroticism and low extroversion in migraine patients predispose them to experience negative emotions and limited social interactions, which increases the risk of depression and reduces resilience to stress [25].

Patients with migraine combined with medication overuse headache (MOH) display lower levels of conscientiousness than those with migraine alone, and female migraine patients combined with MOH show significantly lower levels of extraversion, openness, and responsibility than those with migraine only, whereas no significant differences are observed in male patients, suggesting that gender may modulate the impact of personality traits on migraine [26,27]. Prospective studies have found no significant baseline personality trait differences between migraine patients who develop MOH and those who do not, possibly due to insufficient sensitivity in the simplified BFM or because personality traits may not predict the development of MOH [28].

Personality traits influence susceptibility to mental disorders; for example, neuroticism strongly predicts anxiety, depression, and substance abuse [29]. Thus, early assessment of personality traits may help identify psychopathological risk, enabling timely intervention.

Mental Disorders and Migraine

Mental disorders are syndromes that cause clinically significant impairments in cognition, emotion regulation, or behavior, which can result from the interaction of multiple psychological factors, reflecting dysfunctions in psychological, biological, or developmental processes [30].

Migraine patients often have comorbid mental disorders, which are associated with more frequent migraine attacks, increased medication use, and a higher risk of chronic migraine [31]. Additionally, mental disorders contribute to a reduced quality of life in migraine patients by exacerbating migraine-related disability and impaired occupational functioning, further aggravating the socioeconomic impact of the condition [32].

The prevalence of obsessive-compulsive, avoidant, dependent, and passive-aggressive personality disorders is high among patients with migraine [33]. These personality disorders negatively affect both the course and treatment of migraine and are associated with the development of MOH [34]. The number of personality disorders is positively correlated with the score of the Migraine Disability Assessment Scale (MIDAS), and the presence of multiple comorbidities can worsen migraine severity and impair social functioning.

Migraine patients frequently suffer from depression, anxiety, bipolar disorder, and panic disorder. These mood disorders exacerbate pain, anticipation, fear, and avoidance behaviors, and in turn, migraine can serve as an indicator of the severity of bipolar disorder. Moreover, the presence of migraine can either maintain or exacerbate anxiety and depressive symptoms [32,34,35]. The research using the Minnesota Multiphasic Personality Inventory (MMPI) shows that migraine patients score higher in hypochondriasis, paranoia, histrionics, depression, hypomania, psychasthenia, schizophrenia, and social withdrawal. Chronic migraine patients show a higher tendency for “emotionally inhibited, somatized reactions,” which is consistent with their high harm avoidance, as well as increased levels of depression, introversion, and hypochondriasis. These findings suggest that psychopathological factors are closely associated with the chronicity of migraine [36].

Unlike personality traits, which are normal aspects of an individual's personality, personality disorders are pathological conditions characterized by extreme and rigid personality traits. The high prevalence of personality disorders in migraine patients may be associated with the following pathophysiologic mechanisms. Migraine patients exhibit large iron deposits in the periaqueductal gray matter area (PGA), suggesting abnormal activity in this region [37]. The PGA is connected to the trigeminal caudal nucleus, which plays a critical role in endogenous pain control. Additionally, the PGA also has extensive connectivity with the cingulate gyrus, which influences behavioral changes [38]. The anterior cingulate cortex also plays a role in regulating pain-related emotions [39]. Furthermore, the expression of nitrogen oxides and their associated proinflammatory factors (e.g., IL-1, IL-6) is upregulated, not only in association with migraine onset but also in a way that leads to obsessive-compulsive-like behaviors [40].

**Figure 1:**
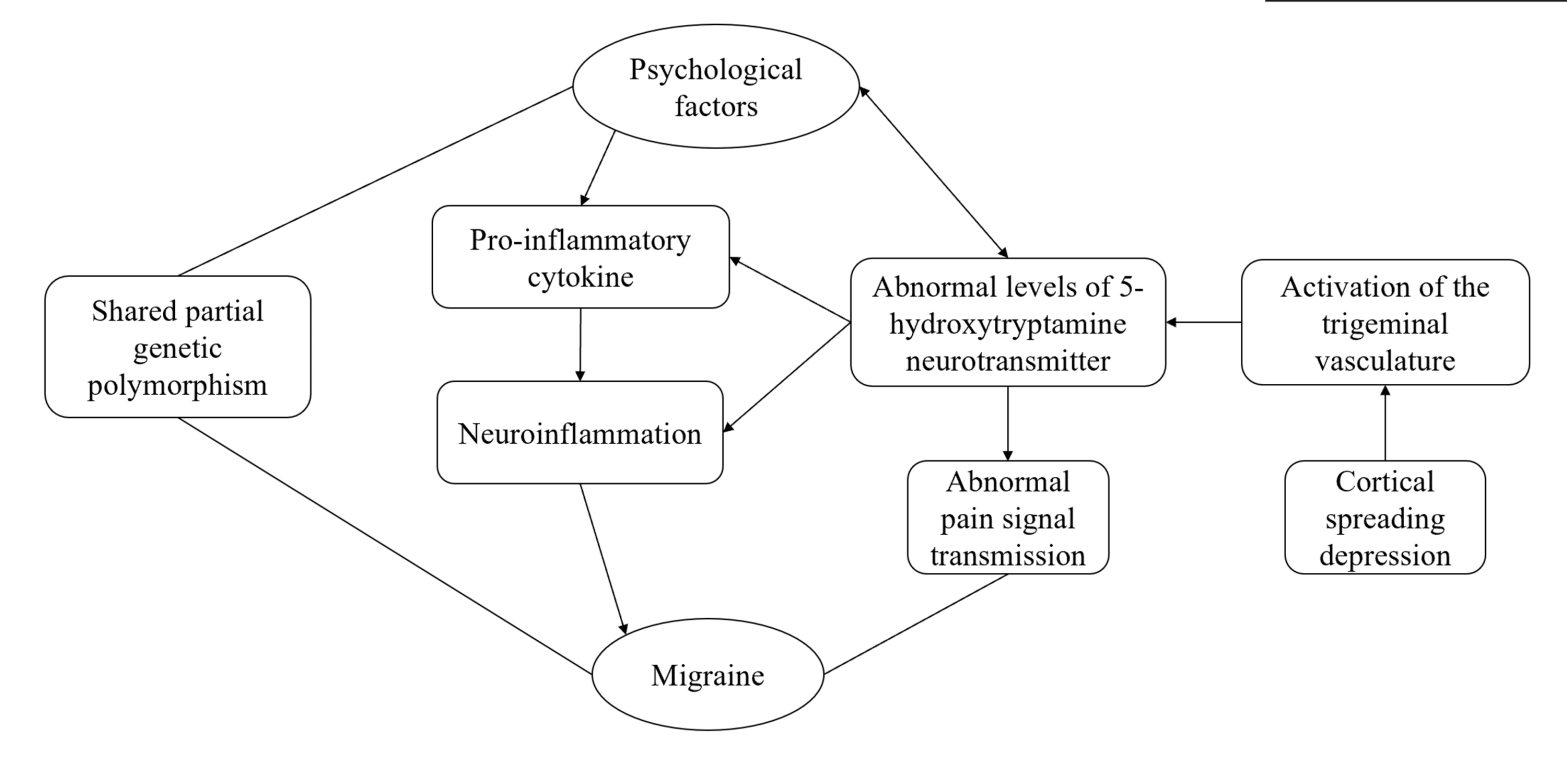
Association of pathophysiologic mechanisms of migraine and psychological factors This diagram provides a simplified illustration of the pathophysiological relationship mentioned in this review between psychological factors and migraine. Psychological factors (e.g., depression) share certain genetic variations with migraine [41]. Psychological factors (e.g., harm avoidance, depression, anxiety, irritability) are associated with abnormal 5-hydroxytryptamine levels, thereby influencing the release of pro-inflammatory cytokines, the onset of neuroinflammation, and altered pain signal processing [23,42]. Moreover, psychological factors (e.g., neuroticism and depression) contribute to neuroinflammation through the release of pro-inflammatory cytokines, which is a key mechanism in migraine pathogenesis [43]. Additionally, cortical spreading depression activates the trigeminovascular system, which, in turn, modulates 5-hydroxytryptamine release by serotonergic neurons, ultimately contributing to migraine onset [24].

Migraine shares approximately 20% of its genetic variance with depression, and genome-wide association studies have indicated that migraine shares a genetic background with several mental disorders [41]. Mood states such as depression, anxiety, and irritability affect pain signaling from the thalamus to the cortex, mediated by 5-HT, norepinephrine, and dopamine. Hypothalamic activation has been linked to neural circuits influenced by migraine prodromes and fear [42]. Inflammatory factors produced by the activation of microglial cells in the insula during depression can lead to neurological inflammation, further triggering central sensitization and pain chronicity, which are key factors in the pathophysiology of migraine [43]. These pathological mechanisms help explain the close association of migraine with psychiatric disorders (Figure 1).

The causal relationship between migraine and mental disorders remains inconclusive. One perspective suggests that recurrent severe headaches contribute to the development of anxiety and depression through cognitive-behavioral patterns such as catastrophizing and pain avoidance [44]. Conversely, another view posits that negative psychological factors increase susceptibility to migraine [22]. It has also been suggested that there may be a bidirectional relationship between migraine and psychiatric disorders [41]. While stronger evidence confirming a causal relationship between migraine and mental disorders is still needed, it is clear that mismanagement of mental disorders undoubtedly increases the risk of individuals with migraine experiencing more frequent and severe headache episodes, as well as a higher risk of migraine-associated disability, through central neurochemical pathways.

Cognitive Processes and Migraine

Thoughts, beliefs, attributions, and attitudes in response to the environment are collectively known as cognitive processes. These cognitive processes play a crucial role in headache and headache-related disability [6]. The following sections provide a detailed exploration of specific cognitive factors, including locus of control beliefs, self-efficacy, anxiety sensitivity, and pain catastrophizing.

Locus of control beliefs reflects an individual's perception of the extent to which an event is controllable. A lower sense of control is associated with more negative emotional, behavioral, and physiological responses. In contrast, a high sense of control has been linked to better treatment outcomes for headaches and lower levels of headache-related disability [6]. Self-efficacy refers to an individual's confidence in their ability to achieve goals through their actions. Individuals with low self-efficacy are more susceptible to autonomic arousal under stress, which increases the risk of headaches. Self-efficacy has also been used as a baseline measure to predict the effectiveness of medication [6,45].

Anxiety sensitivity and pain catastrophizing are cognitive beliefs that affect an individual's self-efficacy and sense of control and play an important role in migraine [46]. Anxiety sensitivity refers to an individual's fear of negative physical, cognitive, and social consequences triggered by anxiety. It is associated with a heightened fear of pain, low pain tolerance, and pain-avoidance behaviors and is strongly associated with more frequent pain episodes, longer headache duration, greater headache intensity, and increased disability in migraine patients. Chronic migraine sufferers have higher anxiety sensitivity scores compared to those with episodic migraine and tend to avoid moderate- to high-intensity physical activities [47,48].

Anxiety sensitivity amplifies negative emotions and pain perception and is strongly associated with the development and persistence of anxiety symptoms [49]. It connects mood disorders to migraine and may contribute to the comorbidity of migraine with anxiety and depression [46]. The association between headache and anxiety sensitivity tends to be more persistent than that with depression and anxiety symptoms [47]. High anxiety sensitivity amplifies the response to stress, greatly increasing the likelihood of a migraine attack [50].

Furthermore, high anxiety sensitivity is linked to more pain avoidance behaviors (e.g., overuse of pain medication, and avoidance of exercise). Patients with high anxiety sensitivity gradually reinforce avoidance behaviors due to anxiety and fear associated with pain avoidance, leading to the persistence of such avoidance behaviors [51]. The cognitive attention dimension of anxiety sensitivity is strongly associated with the overuse of pain medication by patients with migraine [46]. Therefore, the cognitive dimension of anxiety sensitivity may serve as a promising risk indicator. Early identification and intervention for high anxiety sensitivity may be more effective than focusing solely on mood disorders, improving pain perception, and better managing migraine patients, as well as reducing the risk of MOH occurrence.

In the experience of pain, the tendency to “catastrophize” can amplify distress and emotional stress. Pain catastrophizing refers to an individual's processing of pain signals at both the affective and cognitive levels. When emotional and cognitive responses to pain are overly negative, the pain experience is exacerbated, which in turn impacts overall mood and coping abilities. Pain catastrophizing consists of three types of negative psychological responses: difficulty suppressing pain-related thoughts (rumination), exaggerating the negative consequences of pain (magnification), and negative evaluations of one's own coping abilities (helplessness) [52]. This is a common cognitive pattern in migraine patients and is the target of intervention in cognitive behavioral therapy.

Approximately one-quarter of migraine patients meet the criteria for clinical catastrophizing, and higher scores on catastrophizing are associated with increased migraine days, prolonged attack durations, and lower self-efficacy ratings. [52]. Increased rumination and helplessness are positively correlated with migraine intensity [53]. Clinically significant levels of pain catastrophizing increase the risk of chronic migraine. Chronic migraine patients report higher levels of catastrophizing, more severe helplessness, and more frequent rumination than episodic migraine patients [7]. Rumination and magnification reflect fear of pain, while helplessness indicates an underestimation of one’s ability to manage pain [54]. Migraine patients often have difficulty managing pain due to a lack of effective coping strategies, and high levels of pain catastrophizing can lead to pain-avoidant behaviors [53]. Migraine patients' inhibitory control is negatively correlated with levels of pain catastrophizing. As inhibitory control decreases, catastrophizing levels increase, and pain intensity becomes higher. Since high levels of pain catastrophizing are associated with high levels of inhibitory behaviors, this leads to increased pain avoidance behaviors [55]. Additionally, many migraine sufferers experience mild to severe impairments in short- and long-term verbal episodic memory and inhibitory control, the severity of which is positively correlated with the number of medications used, suggesting impaired behavioral control and inhibitory responses [56].

Inhibitory control-related brain regions overlap with prefrontal pain-processing areas. Higher levels of pain catastrophizing correlate with abnormalities in these regions and are associated with increased frontal white matter high-signal volume, decreased white matter integrity, and decreased gray-white matter volume [55]. The orbital frontal cortex, amygdala, and hippocampus play key roles in regulating pain-related emotions and thoughts, and the periaqueductal gray matter and rostral ventral medial medulla are key components of migraine that are linked to these regions, suggesting that migraineurs' brains modulate pain through cognitive processes [57].

In conclusion, negative cognitive beliefs are closely related to the development, progression, and treatment outcomes of migraine. Therefore, nonpharmacological treatments for migraine should not only intervene in mood disorders but should also focus on the patient's extreme sensory processing patterns and coping strategies for abnormal pain perception.

Psychological interventions in migraine treatment

Acute-phase treatment involves the use of nonsteroidal anti-inflammatory drugs (NSAIDs) and specific medications such as triptans and ditans, while prophylactic treatment aims to reduce the frequency of attacks and decrease the intensity and duration of pain through medications such as topiramate, propranolol, metoprolol, amitriptyline, and gabapentin, or the use of botulinum toxin type A injections. Additionally, treatments include calcitonin gene-related peptide receptor (CGRP) antagonists and CGRP monoclonal antibodies. Non-pharmacologic interventions for migraine also include physical and psychotherapy.

Although medications remain the main treatment, in patients with comorbid psychiatric disorders, the use of tricyclic antidepressants may be effective, but in some cases, it can trigger mania in individuals with undiagnosed bipolar disorder, potentially worsening the course of the condition [58]. Some medications (e.g., topiramate) may affect mood and exacerbate the risk of psychiatric disorders [59]. For pregnant and perinatal women, children, and adolescents, treatment options are very limited, and the safety of these treatments requires careful consideration. Moreover, psychological problems in the child and adolescent population are often manifest through physical symptoms (e.g., headaches), and migraine in this population is considered a condition that bridges between pediatrics and psychiatry, resulting in a higher need for psychological interventions [9].

Psychological factors in patients with migraine significantly influence treatment outcomes. Traits such as high levels of neuroticism, depression, and behavioral inhibition have been associated with poor response to CGRP monotherapy [60]. Anxiety and depression have been associated with reduced efficacy of NSAIDs [61]. Personality disorders of type C (avoidant, dependent, and obsessive-compulsive) and anxiety disorders negatively impact the efficacy of erenumab [62]. Dependent personality disorders have also been associated with poor botulinum toxin treatment for botulinum toxin type A and with MOH recurrence [63]. Maladaptive pain coping strategies also affect treatment effectiveness and recurrence [64]. Additionally, factors such as attitudes and beliefs, lack of motivation, poor awareness of triggers, an external locus of control, and low self-efficacy may contribute to poor patient adherence, which in turn can lead to unsatisfactory treatment outcomes [65].

In conclusion, relying solely on pharmacological treatments while neglecting psychological interventions may have a negative impact on the overall management of the disease. Currently, psychotherapy is gradually gaining attention in a variety of forms, such as classical relaxation therapy, biofeedback therapy, cognitive-behavioral therapy, and, more recently, the proposed positive thinking therapy and other forms of meditation exercises [32]. These therapeutic modalities can adjust the patient's perceptions of pain, such as reducing catastrophizing and enhancing self-efficacy, thus controlling medication overuse as well as alleviating the patient's psychiatric comorbidities, and ultimately achieving the goals of improving efficacy, reducing migraine-related disability, improving quality of life, and improving overall prognosis [32]. For patients who have contraindications to applying prophylactic medications and who respond poorly to them, behavioral therapies have been shown to have similar efficacy to prophylactic medications [66]. Neuroimaging studies have confirmed that behavioral therapies, in particular, orthostatic therapies, affect and alter brain regions involved in pain processing and emotion (dorsolateral medial prefrontal cortex, dorsolateral anterior insula, and anterior middle cingulate cortex), with quantifiable effects on migraine-related neural circuits and systems involved in the mechanisms of migraine genesis [67,68]. However, some studies point to the efficacy of current psychological interventions but they do not produce strong effects and do not directly address the effects of trauma and emotional conflict [69]. Meta-analyses have shown small to moderate beneficial effects of psychological interventions on improving migraine frequency, pain intensity, and posttreatment disability, but no beneficial effects on posttreatment quality of life or mood have been found [70]. Some studies have proposed the application of a biopsychosocial model, where the biological level is represented by medication. They argue that optimal individual management occurs when medication effectively prevents the problem, and the individual’s psychosocial environment and behavior are functional. However, if psychological, behavioral, or social dysfunctions are present, the efficacy of medication may be compromised by external triggers and comorbidities [65].

Some studies have proposed the application of a biopsychosocial model, where the biological level is represented by medication. They argue that optimal individual management occurs when medication effectively prevents the problem, and the individual’s psychosocial environment and behavior are functional. However, if psychological, behavioral, or social dysfunctions are present, the efficacy of medication may be compromised by external triggers and comorbidities. Further research is needed to better understand the psychological and headache characteristics of migraine patients and to develop standardized, effective treatment strategies.

## Conclusions

Psychological factors play a key role in the onset, progression, and outcome of migraine. Personality traits, mental conditions, and cognitive progress not only increase susceptibility to migraine but also worsen their chronic migraine.

Personality traits, particularly neuroticism, are often linked to mental disorders and can both raise the risk of migraine and hinder patients' ability to manage pain and stress, leading to chronic migraine. The relationship between mental disorders and migraine is bidirectional: recurring migraine can worsen mood disorders like anxiety and depression, while negative emotions can intensify both the onset and persistence of migraine. Patients with negative cognitive patterns, such as anxiety sensitivity and pain catastrophizing, tend to experience longer pain and engage in avoidant behaviors, like overusing pain medications, which can contribute to chronic migraine.

Therefore, relying solely on medication may not offer complete relief of migraine. Psychological treatments, such as cognitive-behavioral therapy, can help alter negative perceptions of pain, improve coping skills, reduce medication dependence, and lower the risk of chronic migraines, thereby improving the overall prognosis of migraines. Additionally, psychological interventions, including relaxation therapy, biofeedback, positive thinking therapy, and meditation exercises, are increasingly recognized as important complements to pharmacological treatments. However, it is important to note that while psychological treatments have shown promise, their efficacy may vary across individuals, and further research is needed to establish standardized protocols. Additionally, limitations in the current literature, such as small sample sizes or methodological inconsistencies, may affect the generalizability of findings. Despite these limitations, psychological interventions represent a valuable complement to pharmacological treatment in managing migraines.
